# Co-inheritance of Rare Variants of Maturity-Onset Diabetes of the Young (MODY): A Case Report and Review of the Literature

**DOI:** 10.7759/cureus.69039

**Published:** 2024-09-09

**Authors:** Raad Alwithenani, Jehad Alzahrani, Ebtesam Allugmani, Fahad Hakami

**Affiliations:** 1 Department of Medicine, Division of Endocrinology and Diabetes, King Abdulaziz Medical City, Ministry of National Guard Health Affairs, Jeddah, SAU; 2 College of Medicine, King Saud Bin Abdulaziz University for Health Sciences, King Abdullah International Medical Research Center, Jeddah, SAU

**Keywords:** abcc8 gene, blk gene, co-inheritance, hypoglycemia, mody

## Abstract

Maturity onset diabetes of the young (MODY) is a rare, monogenic autosomal dominant form of diabetes that is characterized by early-onset, non-insulin-dependent hyperglycemia, strong family history, and is often misdiagnosed as type 1 or type 2 diabetes. Co-inheritance of multiple MODY genes, however, is rare. We describe here a case of MODY involving co-inherited rare variants in the ABCC8 and B-lymphocyte kinase (BLK) genes. A 55-year-old non-obese man with a past medical history of dyslipidemia and premature ischemic heart disease was initially misdiagnosed with type 2 diabetes for more than 18 years. He is a smoker with a strong family history of diabetes affecting both of his parents and most of his siblings. Despite treatment with different oral antihyperglycemics, his diabetes remained uncontrolled with glycated hemoglobin (HBA1c) between 8 and 10% until the addition of gliclazide, which improved his HBA1c to 5.7%. Based on all the previous information, MODY was suspected, and genetic testing was done, which showed rare variants in the BLK and ABCC8 genes and suggested a co-occurrence of MODY11 and MODY12. This case highlights the importance of accurate genetic testing, which is crucial for proper MODY subtyping, enabling tailored treatment strategies and potentially improving patient outcomes. Moreover, the consistent presence of the BLK gene variant in limited cases of co-inheritance raises questions about its causative role in MODY, suggesting a need for further investigation into its clinical significance.

## Introduction

Maturity onset diabetes of the young (MODY) is an autosomal dominant disorder affecting young people [1]. It is characterized by early onset, typically before the age of 25-35 years, a strong family history of diabetes, usually multigenerational, the absence of pancreatic autoimmunity, which is typical in type 1 diabetes mellitus (T1DM) and latent autoimmune diabetes of adults, and the absence of insulin resistance, which is typical in type 2 diabetes mellitus (T2DM) [2,3]. Interactions of genetic and environmental factors cause most T1DM and T2DM forms and could occur in people under 25 years. The rates of T2DM have been increasing among young people. Nevertheless, T1DM is still the most reported polygenic type of diabetes among this group [4]. The advancement in genetic analysis helped recognize monogenic diabetes [1]. MODY is the most common type of monogenic diabetes, and it results from pathogenic variants in genes encoding proteins responsible for the function of pancreatic beta cells. Based on the gene harboring the causative pathogenic variant, MODY is further divided into 14 subtypes.

Neither the prevalence nor incidence of MODY has been critically explored in the North African and Middle Eastern (MENA) regions, including Saudi Arabia [5], with only a few reports published from Kuwait, Tunisia, Oman, and Qatar [6-9]. In Qatar, the estimated prevalence ranged between 2.2 and 3.4% [9]. The prevalence of MODY in the Western population is approximately 1 in 10,000 adults and 1 in 23,000 children [10]. Almost 90% of MODY cases are caused by pathogenic variants in the glucokinase (GCK) gene (MODY2), HNF1A gene (MODY3), and HNF4A gene (MODY1) [11]. MODY12, caused by pathogenic variants in the ATP-sensitive potassium channel (ABCC8) gene, and MODY11, caused by pathogenic variants in the B-lymphocyte kinase (BLK) gene, were estimated to account for 1.5% and <1% of all the cases across several cohorts [12-15].

## Case presentation

A 55-year-old non-obese man with a history of ischemic heart disease (IHD), T2DM, and dyslipidemia presented to an outpatient diabetes clinic in Jeddah, Saudi Arabia. Premature IHD was diagnosed at the age of 44 years. T2DM was diagnosed at the age of 38 years after having symptoms of polyuria and polydipsia. He was a smoker and not compliant with self-monitoring of blood sugar (SMBG), as well as diet and exercise recommendations. He has no history of diabetic ketoacidosis or hyperglycemic hyperosmolar state. He has no history of microvascular diabetic complications. Our patient has a strong family history of T2DM (father, mother, three brothers, and two sisters), and he has no family history of IHD. The patient does not have any details of his family's diabetes history. In the years prior to his visit to our clinic, the patient had an HbA1c ranging from 8% to 10%, and his LDL levels consistently remained above the optimal target of <1.4 mmol/L. No albumin/creatinine ratio tests were conducted during this period. His treatment regimen included oral antihyperglycemic medications, specifically metformin 1 g once daily (OD) and empagliflozin 12.5 mg OD, as well as atorvastatin 20 mg OD for dyslipidemia. Subsequently, gliclazide MR 90 mg OD was added to his treatment plan, resulting in better blood glucose control. However, this adjustment led to hypoglycemic episodes. When the patient sought care at a primary healthcare facility, the attending physician recommended reducing the gliclazide dose from 90 mg to 60 mg to manage the hypoglycemia.

On examination, the patient's weight was 49 kg, height was 165cm (BMI 18 Kg/m^2^), blood pressure was 115/55 mm/Hg, pulse rate was 89, and body temperature was 36.1˚C. Fasting blood sugar (FBS) was 4.8 mmol/dl. Thyroid, chest, cardiovascular system, abdomen, and foot examinations were normal.

Laboratory results showed glycated hemoglobin (HbA1c) within the target-controlled range (< 7%), indicating tight diabetes control, further supported by his low FBS. However, the lipid profile, especially LDL, was above his optimal target, suggesting uncontrolled dyslipidemia, as seen in Table 1. Unfortunately, the C peptide and antibodies against the pancreatic beta cells were not measured.

**Table 1:** Laboratory values after initial management Hgb: Hemoglobin, HbA1c: glycated hemoglobin, TC: total cholesterol, TG: triglyceride, LDL: low-density lipoprotein

Parameter	Range	Result
Hgb	13 – 18 g/dL	16.1 g/dL
HbA1c	3.9 – 5.7 %	5.70%
TC	< 5.18 mmol/L	5.21 mmol/L
TG	< 1.7 mmol/L	1.63 mmol/L
LDL	optimal: < 1.4 mmol/L	3.06 mmol/L

A management plan included decreasing the gliclazide dose to 30 mg, continuing metformin 1g OD, empagliflozin 12.5 mg OD, atorvastatin 40 mg with the addition of ezetimibe 10 mg, aspirin 81 mg, reinforcing lifestyle change in the form of a healthy diet and regular exercise, and SMBG. In addition, we referred him to cardiology, ophthalmology, diabetic education, and a dietitian. Given his clinical profile, including the age of diagnosis, strong multigenerational family history of diabetes, low BMI, and sensitivity to sulfonylurea (SU), genetic testing was ordered to define the type of diabetes in this patient. Exome sequencing was performed by a reference that identified heterozygous variants of uncertain significance in the ABCC8 and BLK genes, suggesting MODY12 and MODY11, respectively. 

After two months, the patient came to the clinic. He was doing well, with no more hypoglycemia after decreasing the dose of gliclazide and complying with his new regimen. Nevertheless, the patient was recommended to bring family members for a genetic screening; however, he refused initially and was lost to follow-up after that.

## Discussion

MODY is a condition generally marked by early onset, autosomal dominant inheritance, and the absence of β-cell autoimmunity, with the pancreatic β-cell typically functioning normally [16]. Fourteen distinct gene mutations have been identified to date, including six genes that encode proteins associated with subtypes 1 through 6: hepatocyte nuclear factor 4α (HNF4α), glucokinase (GCK), hepatocyte nuclear factor 1α (HNF1α), pancreatic and duodenal homeobox 1 (PDX1), hepatocyte nuclear factor 1β (HNF1β), and neurogenic differentiation 1 (NEUROD1) [17-20]. Genetic tests are currently available tools to diagnose MODY and help in offering appropriate treatment [16]. Genetic testing should be offered for non-obese individuals with hyperglycemia who exhibit no signs of β-cell autoimmunity, maintain β-cell function, and have a strong family history of diabetes, particularly among first-degree relatives [16]. Our patient was diagnosed with diabetes in his 30s and has a strong family history of type 2 diabetes in his parents and siblings, low BMI, as well as he responded well to gliclazide. These clinical data raised the suspicion of MODY in our patient.

 In our patient, genetic testing showed a heterozygous variant of uncertain significance in the genes ABCC8 and BLK. The ABCC8 variant (NM_000352.6:c.2993G>C (p.Arg998Pro), dbSNP: rs776248166) has been previously mentioned in two diabetes-related studies but without detailed information [21,22]. It has a frequency of 0.0008% [2/248,040 chromosomes] based on the Genome Aggregation Database (gnomAD; https://gnomad.broadinstitute.org). Unfortunately, genetic testing was not done on the remaining affected family members. Therefore, segregation analysis was not performed, and its classification remained a variant of uncertain significance (VUS). The same applies to the BLK variant (NM_001715.3):c.575G>A (p.Arg192Gln), dbSNP: rs761710037), which has a frequency of 0.001% (3/250,876 chromosomes) based on the Genome Aggregation Database but has never been described in any published work, to the best of our knowledge.

Pathogenic variants in the ABCC8 gene impair the encoded ABCC8 protein, which regulates glucose-dependent insulin secretion in pancreatic beta cells [23]. In MODY11, the BLK protein in upregulating the transcription factors PDX1 and NKX6.1 is affected, subsequently impairing their role in enhancing pancreatic beta-cell mass as well as insulin synthesis and secretion [24].

Since most cases of MODY are misdiagnosed as type 1 or type 2 diabetes, a combination of both laboratory and clinical findings is needed to diagnose patients with the disease [16]. When we come to clinical findings, The clinical phenotype of the ABCC8 gene (MODY 12) might vary depending on the type and location of the mutation, as has been found [12]. Even among members of the same family, according to an observational study [25]. Clinical manifestations may start at any age, including infancy with hyperinsulinemic hypoglycemia or neonatal DM and childhood or adulthood with overt DM [12]. Timmers et al. reported a 35-year-old woman who was diagnosed with (MODY 12) and underwent a lower limb amputation due to severe Charcot arthropathy secondary to diabetic microvascular complications [26]. The previous report may indicate that MODY 12 is not always harmless and can be associated with progressive diabetic complications. According to a systematic review of non-neonatal DM secondary to ABCC8, around 50% of patients with ABCC8 variants developed diabetic retinopathy, and the ABCC8 variant may be responsible for the progression to proliferative retinopathy [27]. Given that our patient had T2DM for about seven years prior to the diagnosis of premature IHD, it might suggest that the ABCC8 gene, in addition to smoking and dyslipidemia, may have a role in the progression of IHD. Patients with ABCC8 have been found to be highly sensitive to SU therapy, and it has since been recommended as the first line of treatment for the ABCC8 gene, according to two systematic reviews [27,28]. Our patient showed an excellent response to the lowest dose of the extended-release gliclazide, in addition to the MTF and Dapagliflozin.

Very few cases have been reported for the BLK gene (MODY 11), which was initially identified in 2009 in three families by Borowiec et al. [24]. Clinical manifestations of this type include overweight and overt diabetes in adulthood [15]. However, there was a recent case report of a 17-year-old male who was first diagnosed with T1DM before the BLK gene was genetically identified, and he required insulin and SU to control his condition [29]. Given all of these, Bonnefond et al. concluded that MODY is not likely to be associated with the BLK-p-A71T mutation [30]. In addition, a recent study analyzed the genetic evidence at the variation and gene levels for the involvement of BLK in MODY. Their findings showed that the BLK gene is not a cause of MODY and even recommended removing the BLK gene from the genetic testing [31]. In their systematic review of MODY treatment, Delvecchio et al. recommended insulin, OAHs, and diet as treatments for patients with the BLK gene, which was supported in the previous case report [28]. However, our patient had a normal BMI and showed an excellent response to only OAH, despite the poor compliance to diet and exercise and didn’t require any insulin.

To our knowledge, there are only a few cases with co-inheritance of two MODY genes, the genetic testing for our patient has shown a heterozygous variant of uncertain significance in both genes ABCC8 and BLK, this is the only reported case with (MODY 11 and 12) co-Inheritance. However, we found two additional reported cases with MODY co-inheritance. First, a reported case in Germany of a 38-year-old African male presented with ketosis-prone diabetes was found to have a co-inheritance of the PAX4 gene (MODY 9) and BLK gene (MODY 11) [32]. It should be noted that previous reports have linked PAX4 to ketosis-prone diabetes [33], suggesting that the PAX4 gene (MODY 9) is the primary gene responsible for the patient's presentation and that BLK may or may not be involved.

In the other report from China, a 27-year-old male with a past surgical history of duodenal atresia at neonatal age was also found to have co-inheritance with the HNF-1b gene (MODY 5) and the BLK gene (MODY 11). Following the MODY diagnosis, the patient required very intensive insulin therapy to control his blood sugar. The report also revealed evidence of bilateral renal cysts on ultrasound imaging. Based on the multisystem phenotype and the known “renal cyst and diabetes syndrome”, HNF-1b gene (MODY 5) seems to be the most likely responsible gene for this patient presentation, where the BLK mutation effect was difficult to evaluate in this case, according to the author [34].

Our patient had a normal BMI, progressive diabetic complication in the form of IHD, and responded very well to a small dose of SU; the ABCC8 mutation phenotype matches the clinical presentation of our patient rather than the BLK mutation phenotype, where the BLK gene role is again difficult to evaluate its effect all these reports, including ours, which can support the findings that have been suggested by Bonnefond et al. and Laver et al. [30,31]. Since the BLK gene mutation is common in the population and lacks rarity, the authors suggested that it does not cause MODY.

## Conclusions

In conclusion, we present a MODY case with co-inherited rare variants in the BLK and ABCC8 genes. While the ABCC8 variant is linked to diabetes, the BLK variant has not been previously described. The presence of the BLK gene (MODY 11) in a few cases of co-inheritance suggests that it may not cause MODY and could be more common than initially thought. Further research is needed to clarify the role of the BLK gene in MODY. Moreover, offering genetic testing for suspected MODY cases is crucial for accurate diagnosis, appropriate treatment, and preventing complications.

## References

[1] Amed S, Oram R (2016). Maturity-onset diabetes of the young (MODY): Making the right diagnosis to optimize treatment. Can J Diabetes.

[2] McDonald TJ, Ellard S (2013). Maturity onset diabetes of the young: identification and diagnosis. Ann Clin Biochem.

[3] Peixoto-Barbosa R, Reis AF, Giuffrida FM (2020). Update on clinical screening of maturity-onset diabetes of the young (MODY). Diabetol Metab Syndr.

[4] Patterson C, Guariguata L, Dahlquist G, Soltész G, Ogle G, Silink M (2014). Diabetes in the young - a global view and worldwide estimates of numbers of children with type 1 diabetes. Diabetes Res Clin Pract.

[5] Elkholy S, Lardhi AA (2015). Do we need to test for maturity onset diabetes of the young among newly diagnosed diabetics in Saudi Arabia?. Int J Diabetes Mellit.

[6] Al-Kandari H, Al-Abdulrazzaq D, Davidsson L, Al-Mulla F (2020). Maturity-onset diabetes of the young (MODY): a time to act. Lancet Diabetes Endocrinol.

[7] Ben Khelifa S, Martinez R, Dandana A, Khochtali I, Ferchichi S, Castaño L (2018). Maturity onset diabetes of the young (MODY) in Tunisia: low frequencies of GCK and HNF1A mutations. Gene.

[8] Woodhouse NJ, Elshafie OT, Al-Mamari AS, Mohammed NH, Al-Riyami F, Raeburn S (2010). Clinically-defined maturity onset diabetes of the Young in Omanis: absence of the common Caucasian gene mutations. Sultan Qaboos Univ Med J.

[9] Elashi AA, Toor SM, Diboun I (2022). The genetic spectrum of maturity-onset diabetes of the young (MODY) in Qatar, a population-based study. Int J Mol Sci.

[10] Owen K (2023). Orphanet: MODY. https://www.orpha.net/en/disease/detail/552.

[11] Kleinberger JW, Pollin TI (2015). Undiagnosed MODY: time for action. Curr Diab Rep.

[12] Riveline JP, Rousseau E, Reznik Y (2012). Clinical and metabolic features of adult-onset diabetes caused by ABCC8 mutations. Diabetes Care.

[13] Donath X, Saint-Martin C, Dubois-Laforgue D (2019). Next-generation sequencing identifies monogenic diabetes in 16% of patients with late adolescence/adult-onset diabetes selected on a clinical basis: a cross-sectional analysis. BMC Med.

[14] Park SS, Jang SS, Ahn CH (2019). Identifying pathogenic variants of monogenic diabetes using targeted panel sequencing in an East Asian population. J Clin Endocrinol Metab.

[15] Naylor R, Johnson AK, Del Gaudio D (2018). Maturity-Onset Diabetes of the Young Overview. https://www.ncbi.nlm.nih.gov/books/NBK500456/.

[16] Urakami T (2019). Maturity-onset diabetes of the young (MODY): current perspectives on diagnosis and treatment. Diabetes Metab Syndr Obes.

[17] Kavvoura FK, Owen KR (2012). Maturity onset diabetes of the young: clinical characteristics, diagnosis and management. Pediatr Endocrinol Rev.

[18] Kim SH (2015). Maturity-onset diabetes of the young: what do clinicians need to know?. Diabetes Metab J.

[19] Thanabalasingham G, Pal A, Selwood MP (2012). Systematic assessment of etiology in adults with a clinical diagnosis of young-onset type 2 diabetes is a successful strategy for identifying maturity-onset diabetes of the young. Diabetes Care.

[20] Hattersley AT, Greeley SA, Polak M (2018). ISPAD Clinical Practice Consensus Guidelines 2018: the diagnosis and management of monogenic diabetes in children and adolescents. Pediatr Diabetes.

[21] Ittisoponpisan S, David A (2018). Structural biology helps interpret variants of uncertain significance in genes causing endocrine and metabolic disorders. J Endocr Soc.

[22] Kuznetsova KG, Vašíček J, Skiadopoulou D (2024). Bioinformatics pipeline for the systematic mining genomic and proteomic variation linked to rare diseases: the example of monogenic diabetes. PLoS One.

[23] Ashcroft FM (2005). ATP-sensitive potassium channelopathies: focus on insulin secretion. J Clin Invest.

[24] Borowiec M, Liew CW, Thompson R (2009). Mutations at the BLK locus linked to maturity onset diabetes of the young and beta-cell dysfunction. Proc Natl Acad Sci U S A.

[25] Kapoor RR, Flanagan SE, James CT (2011). Hyperinsulinaemic hypoglycaemia and diabetes mellitus due to dominant ABCC8/KCNJ11 mutations. Diabetologia.

[26] Timmers M, Dirinck E, Lauwers P, Wuyts W, De Block C (2021). ABCC8 variants in MODY12: review of the literature and report of a case with severe complications. Diabetes Metab Res Rev.

[27] Li M, Han X, Ji L (2021). Clinical and genetic characteristics of ABCC8 nonneonatal diabetes mellitus: a systematic review. J Diabetes Res.

[28] Delvecchio M, Pastore C, Giordano P (2020). Treatment options for MODY patients: a systematic review of literature. Diabetes Ther.

[29] Bulus AD, Yasartekin Y, Ortlek H, Ceylan AC (2023). BLK-1 mutation with maturity onset diabetes of the young 11: a case report. Ann Clin Case Rep.

[30] Bonnefond A, Yengo L, Philippe J (2013). Reassessment of the putative role of BLK-p.A71T loss-of-function mutation in MODY and type 2 diabetes. Diabetologia.

[31] Laver TW, Wakeling MN, Knox O (2022). Evaluation of evidence for pathogenicity demonstrates that BLK, KLF11, and PAX4 should not be included in diagnostic testing for MODY. Diabetes.

[32] Schmidt W, Lankers H (2016). Co-inheritance of PAX4 and BLK mutations (MODY 7 and 9) in a 38-year-old African patient with ketosis-prone diabetes. Endocr Abstr.

[33] Mauvais-Jarvis F, Smith SB, Le May C (2004). PAX4 gene variations predispose to ketosis-prone diabetes. Hum Mol Genet.

[34] Du T, Zeng N, Wen X, Zhu P, Li W (2020). A rare combination of MODY5 and duodenal atresia in a patient: a case report. BMC Med Genet.

